# Comparative TMT Proteomic Analysis Unveils Unique Insights into *Helicoverpa armigera* (Hübner) Resistance in *Cajanus scarabaeoides* (L.) Thouars

**DOI:** 10.3390/ijms22115941

**Published:** 2021-05-31

**Authors:** Abigail Ngugi-Dawit, Isaac Njaci, Thomas J. V. Higgins, Brett Williams, Sita R. Ghimire, Sagadevan G. Mundree, Linh Thi My Hoang

**Affiliations:** 1 Centre for Agriculture and the Bioeconomy (CAB), Queensland University of Technology (QUT), Brisbane 4001, Australia; b.williams@qut.edu.au; 2 Biosciences Eastern and Central Africa—International Livestock Research Institute (BecA-ILRI) Hub, P.O. 30709, Nairobi 00100, Kenya; injaci@cgiar.org (I.N.); s.ghimire@cgiar.org (S.R.G.); 3 Commonwealth Scientific and Industrial Research Organisation (CSIRO), Agriculture and Food, Canberra 2601, Australia; Tj.Higgins@csiro.au

**Keywords:** *Cajanus cajan*, ICPL 87, IBS 3471, pigeonpea, TMT, phenypropanoid pathway, quercetin, feeding bioassays

## Abstract

Pigeonpea [*Cajanus cajan* (L.) Millspaugh] is an economically important legume playing a crucial role in the semi-arid tropics. Pigeonpea is susceptible to *Helicoverpa armigera* (Hübner), which causes devastating yield losses. This pest is developing resistance to many commercially available insecticides. Therefore, crop wild relatives of pigeonpea, are being considered as potential sources of genes to expand the genetic base of cultivated pigeonpea to improve traits such as host plant resistance to pests and pathogens. Quantitative proteomic analysis was conducted using the tandem mass tag platform to identify differentially abundant proteins between IBS 3471 and ICPL 87 tolerant accession and susceptible variety to *H. armigera,* respectively. Leaf proteome were analysed at the vegetative and flowering/podding growth stages. *H. armigera* tolerance in IBS 3471 appeared to be related to enhanced defence responses, such as changes in secondary metabolite precursors, antioxidants, and the phenylpropanoid pathway. The development of larvae fed on an artificial diet with IBS 3471 lyophilised leaves showed similar inhibition with those fed on an artificial diet with quercetin concentrations with 32 mg/25 g of artificial diet. DAB staining (3,3′-diaminobenzidine) revealed a rapid accumulation of reactive oxygen species in IBS 3471. We conclude that IBS 3471 is an ideal candidate for improving the genetic base of cultivated pigeonpea, including traits for host plant resistance.

## 1. Introduction

Pigeonpea [*Cajanus cajan* (L.) Millspaugh] is the sixth most important legume grain in the world, representing 5% of world legume production [1]. It is grown in the semi-arid tropics on over seven million hectares across the globe, with annual production approaching six million tonnes per year [2]. Pigeonpea is an economically important crop in supporting people’s livelihoods depending on rain-fed agriculture in the arid and the semi-arid tropics (SAT) [3]. Pigeonpea is rich in protein, carbohydrates, B-group vitamins, amino acids, calcium, fibre, and iron; as a result, it is an important plant-based protein for vegetarians and vegans. Pigeonpea is one of the hosts of the devastating polyphagous insect *Helicoverpa armigera* (Hübner) (Lepidoptera: Noctuidae), commonly known as cotton bollworm or pod borer [4]. *H. armigera* causes substantial economic losses globally and is one of the most devastating pests worldwide. This is due to its extreme polyphagous nature, migratory behaviour, high and rapid fecundity rate, diapause capability, and ability to adapt to a new environment. In addition, it has developed resistance to many commercially available insecticides including *Bacillus thuringiensis* (Bt)—transgenic crops [5]. As a result, modern breeding programs are expanding the genetic base of the cultivated gene pool of pigeonpea by using crop wild relatives (CWRs). The CWRs possess genes for resistance to several stresses such as insect pests, pathogens, and other agronomic constraints [6].

*Cajanus scarabaeoides* (L.) Thouars is a CWR sharing the same chromosome number (2n = 2x = 22) as cultivated pigeonpea. It is sexually compatible with cultivated pigeonpea, resulting in fertile interspecific hybrids [7]. *C. scarabaeoides* is a potential source of host plant resistance to pod borer, pod fly, pod wasp and phytophthora blight [8]. *C. scarabaeoides* has several host resistance mechanisms against pod borer such as antixenosis (non-host preference), antibiosis (harmful effect on insect biology), and non-preferential oviposition contributed by its trichomes and its exudates [8].

Plants have numerous host resistance mechanisms that help defend against attacks from physical injuries either from insect feeding or mechanical wounding [9,10]. When a plant detects an injury, it will initiate defence mechanisms locally (directly at injured sites) or systemically, or both, within a few seconds to hours. These defence mechanisms consist of generation, release, perception, and transduction of internal signals via a complex delivery network to activate the expression and production of multiple defensive compounds such as secondary metabolites [11].

Secondary metabolites play a crucial role in plant protection against microorganisms (viruses, bacteria, fungi), herbivores (vertebrates, arthropods, molluscs), and competing plants (allelopathy). Other functions include use as repellents (quercetin, rutin, tannin) and attractants (anthocyanins, volatile terpenes, phenols, fragrances, colour) to attract enemies and animals for pollination and seed dispersal [12,13]. The phenylpropanoid pathway is one of the most significant branches of secondary metabolism of the aromatic amino acid phenylalanine, except for some monocots which use tyrosine [14]. The phenylpropanoid pathway has five branches, leading to flavonoids, monolignols, phenolic compounds, stilbenes, and coumarins [14]. Plants use flavonoids as a feeding deterrent, growth inhibitor, and toxins that defend against insects such as members of the Lepidoptera, Coleoptera, Hymenoptera, and Hemiptera [14].

Tandem Mass Tag (TMT) are sensitive, quantitative proteomic platforms using isobaric labelling based on tandem mass spectrometry (MS/MS) to simultaneously identify and determine the relative abundances of peptides from different samples [15].

Previously, we reported that leaf extracts from IBS 3471, a *C. scarabaeoides* accession, caused the highest inhibition of *H. armigera* larval growth and development [16]. Larval growth inhibition by IBS 3471 leaf was reduced by heat, indicating that proteins are playing a role. Therefore, we used TMT label-based comparative quantitative analyses of the leaf proteome of two contrasting *Cajanus* spp. (tolerant and susceptible to *H. armigera*), at vegetative and flowering/podding stages. The analysis revealed most of the highly accumulated proteins in *C. scarabaeoides* were involved in ROS scavenging, signalling, and secondary metabolites such as phenylpropanoids.

## 2. Results

### 2.1. MS Analysis Revealed Differences in the Abundance of Hundreds of Proteins between H. armigera Tolerant and Susceptible Genotypes

TMT quantitative proteomic analysis of leaves of IBS 3471, a *C. scarabaeoides* tolerant accession, and ICPL 87, a *C. cajan* susceptible variety, resulted in the identification of 6795 high confident proteins in both growth stages (Supplementary Table S1). Density plot and box plot analysis of normalised data showed similar protein ratio distribution (Supplementary Figure S1).

**Figure 1:**
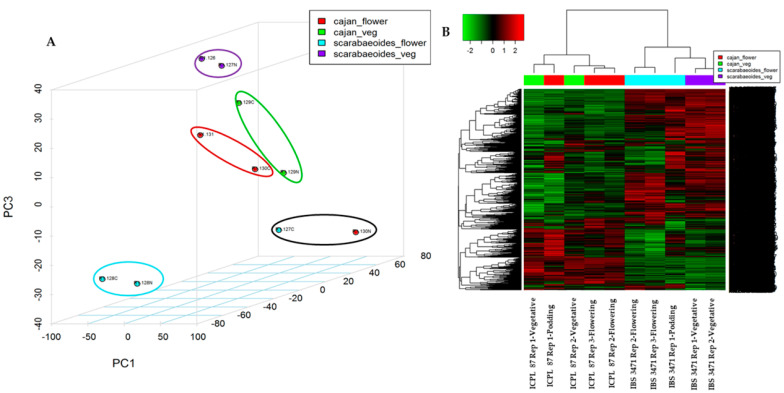
Clustering of leaf proteome of IBS 3471 and ICPL 87 displayed as a 3D PCA and heatmap. (**A**) A 3D Principal Component Analysis (3D PCA) of accumulated proteins of IBS 3471 and ICPL 87. IBS 3471 samples highlighted in a purple circle (126,127N) are at the vegetative stage, blue circle (128C,128N) for the flowering stage, and black circle for the podding stage (127C). For ICPL 87 samples, at vegetative stage are highlighted in a green circle (129N,129C), at the flowering stage in a red circle (131,130C), and a black circle for the podding stage (130N). (**B**) A heat map showing unsupervised hierarchical clustering of statistically significantly differentially abundant proteins for IBS 3471 and ICPL 87.

The 3D Principal Component Analysis (PCA) plot result showed the accumulated proteins clustered together according to growth stages (vegetative or flowering/podding) and genotypes/species (IBS 3471 or ICPL 87). The two samples (126,127N), from the vegetative stage of IBS 3471 clustered together and further away from the samples at the flowering stage (128C,128N) and podding stage (127C) (Figure 1A). ICPL 87 samples showed distinct clustering to IBS 3471 samples. Vegetative samples for ICPL 87 (129N,129C) clustered together and near the flowering stage samples for ICPL 87 (131,130C), while the podding stage sample for ICPL 87 clustered away (130N) and in proximity to IBS 3471 sample at the podding stage (Figure 1A). This PCA clustering of samples represents a high level of correlation between the biological replicates and the distinct responses (tolerance or susceptible) of the two genotypes.

Unsupervised hierarchical clustering of statistically significant, differentially abundant proteins (DAPs) was generated as a heat map (Figure 1B). The various samples segregated into distinct clusters representing proteins exclusively abundant in the individual group of samples. The proteins at highest abundance and accumulation in the wild pigeonpea (IBS 3471) were at reduced levels in the cultivated pigeonpea (ICPL 87), and vice versa (Figure 1B).

**Figure 2:**
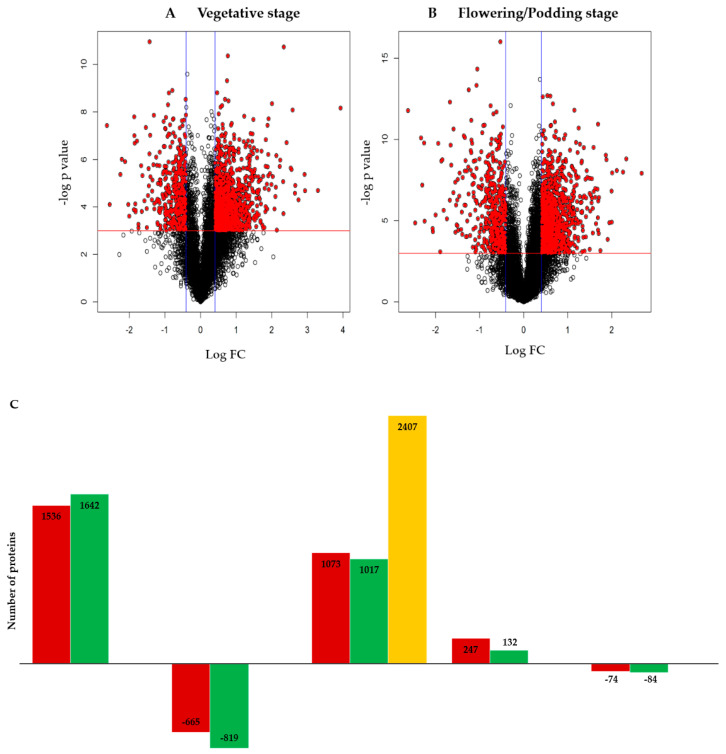
Representation of all identified proteins as a volcano plot and statistical summaries of the differentially abundant proteins (DAPs). (**A**) Volcano plot showing all identified proteins during the vegetative stage. (**B**) Volcano plot showing all identified proteins during flowering/podding stage. Points above the red horizontal line represent proteins with significantly different abundances (*p* < 0.05) and the points below the red horizontal line are not significantly different (*p* < 0.05). All the red point represents proteins with a FC > ±1.5, while all the black points represent proteins with a FC < +1.5. (**C**) Summary of statistically significantly (*p* < 0.05) proteins and differentially abundant proteins (DAPs) identified for the two genotypes at the vegetative and flowering/podding growth stage.

A T-test was done on the identified 6795 proteins to determine significant differences in abundance between the two genotypes (*p* < 0.05) for the overall dataset (combined vegetative and flowering/podding growth stages) (Figure 2A,B). From the overall dataset, 2035 proteins were identified at *p* < 0.05 using Benjamini-Hochberg (BH) adjustment analysis. In comparison, a total of 3785 proteins out of 6795 proteins identified were not significantly different (*p* < 0.05). The significantly different (*p* < 0.05) proteins for vegetative growth stage had 2201 proteins (1536 up-accumulated and 665 down-accumulated) and the flowering/podding stage had 2461 proteins (1642 up-accumulated and 819 down-accumulated) (Figure 2C). The *t*-test data is represented graphically as a volcano plot (-og *p*-value) versus (Log Fold change [FC] of Resistance/Susceptible) (Figure 2A,B). Points above the red horizontal line represent proteins with significantly different abundances at *p* < 0.05. The points below the red horizontal line represent proteins, which are not significantly different at *p* < 0.05 (Figure 2A,B). Additionally, all the red point represents proteins with a fold change (FC) of more than ±1.5, while all the black points represent proteins with a FC of less than ±1.5. Proteins with a fold change (FC of Resistance/Susceptible) >±1.5 that were statistically significant (*p* < 0.05) were regarded as differentially abundant proteins (DAPs). A total of 2407 DAPs were identified in the combined growth stages and 1073 DAPs were identified for the vegetative stage, while for flowering/podding stage there were 1017 DAPs (Figure 2C). The DAPs were further subjected to log_2_FC analysis where the vegetative growth stage had 321 proteins (247 with >1.5 and 74 > −1.5) and the flowering/podding stage had 216 proteins (132 with >1.5 and 84 > −1.5) (Figure 2C).

### 2.2. PANTHER and KEGG GO Classification

**Figure 3:**
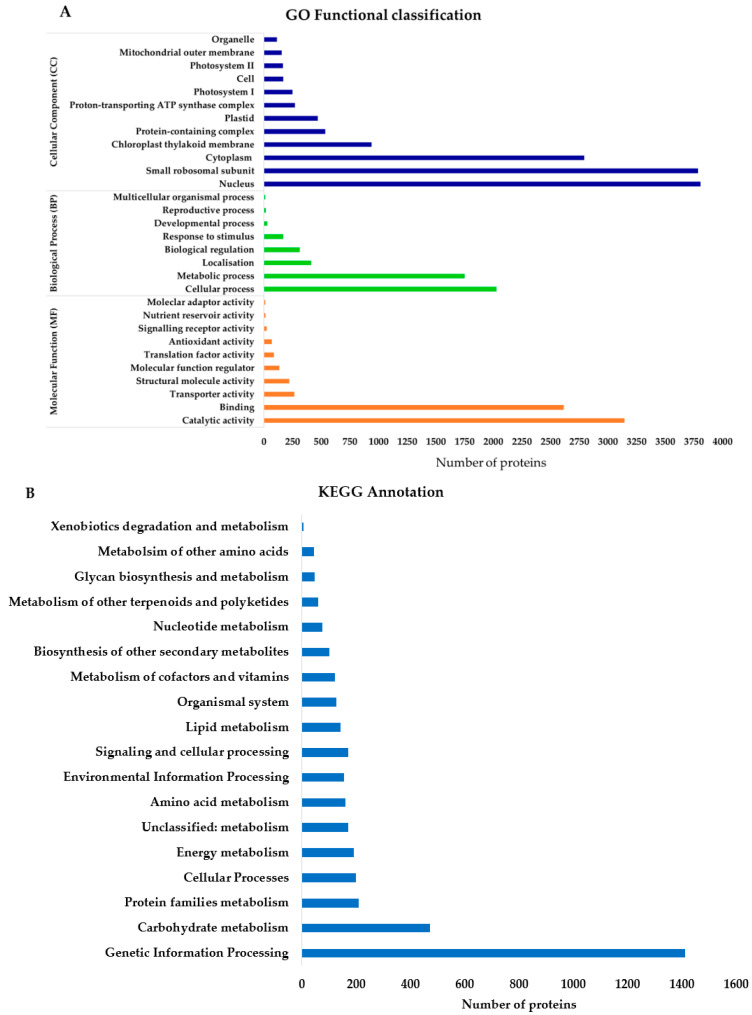

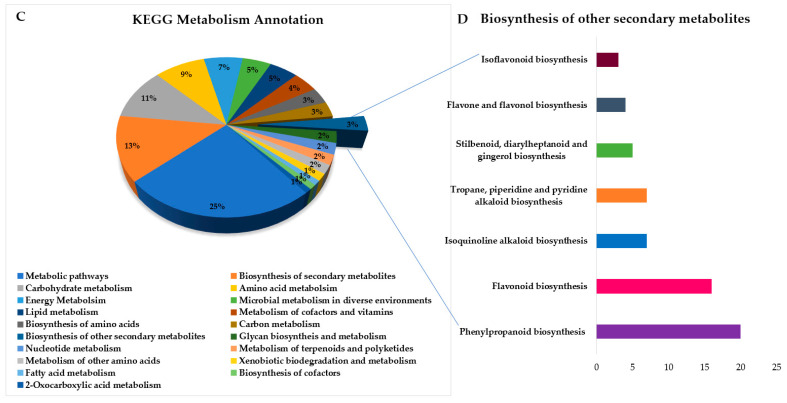
GO functional classification of identified proteins and KEGG annotation of differentially abundant proteins (DAPs). (**A**) PANTHER GO functional classification in terms of biological process, molecular function, and cellular component. (**B**) KEGG total protein annotation. (**C**) KEGG metabolism annotation. (**D**) Biosynthesis of other secondary metabolites annotation.

To further investigate the proteomic changes, the identified proteins were subjected to the PANTHER classification system, resulting in three Gene Ontology (GO) classifications, namely the molecular function (MF), cellular component (CC), and biological process (BP) (Figure 3A).

From the molecular function results, catalytic activity had the highest number of proteins identified (3143 proteins), followed closely by binding (2616 proteins) and transporter activity proteins (264 proteins) (Figure 3A, Supplementary Table S1). For biological processes, cellular processes had the highest number of proteins annotated (2028 proteins) followed by metabolic (1750 proteins) and localisation processes (412 proteins). For cellular component classification, the nucleus had more protein annotated (3807 proteins) followed by small ribosomal subunit (3786 proteins), cytoplasm (2792 proteins), and chloroplast thylakoid membrane (939 proteins) (Figure 3A, Supplementary Table S1).

Besides the PANTHER classification, differentially abundant proteins were analysed by KEGG BlastKOALA (Kyoto University, Kyoto, Japan). Genetic information processing which involves transcription, translation, folding, sorting, degradation, replication, and repair processes had the highest number of annotated proteins. This was followed by carbohydrate metabolism, energy metabolism, amino acid metabolism, and biosynthesis of other secondary metabolites (Figure 3B). Other activities with higher protein annotations were environmental information processing (membrane transport and signal transduction), signalling and cellular processing (involved in relaying signals and changing cellular activity), and other cellular processes (transport and catabolism, cell growth, and death) (Figure 3B).

The KEGG metabolism annotation classified the proteins into several clusters (Figure 3C). Metabolic pathways had the highest number of proteins annotated, followed by carbohydrate metabolism, energy metabolism, lipid metabolism, and biosynthesis of amino acids and other secondary metabolites (Figure 3C).

For the biosynthesis of other secondary metabolites, phenylpropanoid biosynthesis had the highest number of proteins annotated, followed closely by flavonoid biosynthesis (Figure 3D). Other biosyntheses included isoquinoline alkaloid, tropane, piperidine and pyridine alkaloid biosynthesis, stilbenoid, diarylheptanoid and gingerol biosynthesis, flavone and flavonol biosynthesis, and isoflavonoid biosynthesis (Figure 3D).

### 2.3. Majority of the Defence-Related Proteins Were Involved in ROS and Signalling Pathways

Selected differentially abundant proteins (DAPs with >±1.5 FC) were further subjected to log_2_FC analysis and categorised into functional groups such as antioxidant, ROS scavenger, detoxification enzymes, secondary metabolite, and signalling. Enzymes crucial in defence, especially against insects and pathogens such as glutathione-S-transferases (GSTs) and cytochrome P450, were among the detoxifying enzymes identified. ROS in moderate amounts act as signalling molecules in plants, whereas an excessive amount of ROS is detrimental to the plant. Antioxidant and ROS scavengers such as peroxidases, glutathione peroxidase, superoxide dismutase, and peroxiredoxin are vital in maintaining the ROS balance in the plant cells and crucial for plant homeostasis and survival were also identified (Supplementary Table S1). Overall, IBS 3471 had more abundant proteins involved in defence than ICPL 87 (Table 1, Supplementary Table S1).

#### Oxidative Burst Examination of ROS Accumulation on Leaves

**Figure 4:**
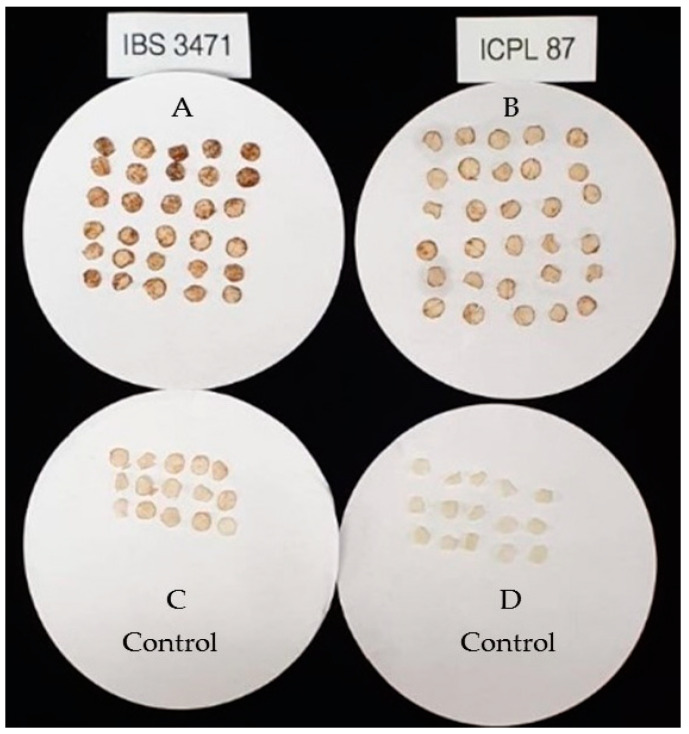
DAB-stained leaf discs to detect the presence of ROS: (**A**) IBS 3471 DAB-stained leaf discs; (**B**) ICPL 87 DAB-stained leaf discs; (**C**) IBS 3471 control leaf discs; (**D**) ICPL 87 control leaf discs.

One of the earliest observable features involved in rapid active plant responses is oxidative burst resulting in the rapid production of a vast amount of ROS. The level of ROS accumulation in the leaves of IBS 3471 and ICPL 87 was assessed using DAB staining. The leaves of IBS 3471 had darker staining, indicating higher ROS accumulation levels than in ICPL 87 leaves (Figure 4).

### 2.4. Quercetin-A Well-Known Insect Inhibitor Was More Abundant in IBS3471 Than in ICPL87 Leaves

**Figure 5:**
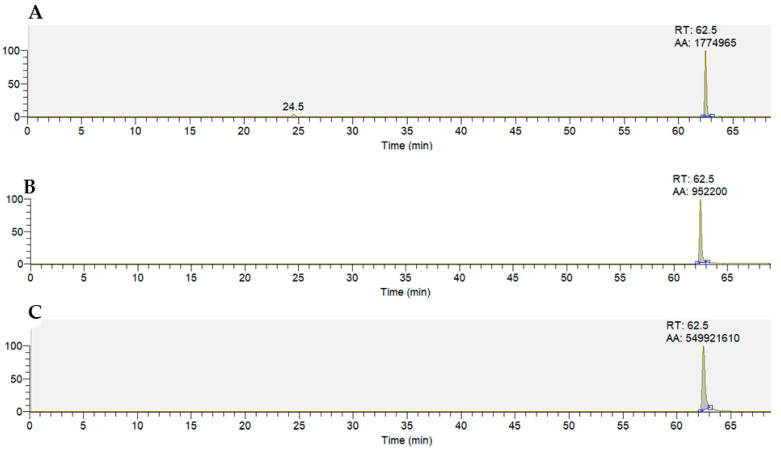
Quercetin LCMS chromatogram observed in sample IBS 3471(**A**), sample ICPL 87 (**B**), and quercetin standard (**C**).

Pigeonpea leaves are rich in different flavonoid sub-classes. The LCMS analysis on methanol extracts from the freeze-dried leaf samples revealed several ion formulas and mass-to-charge (m/z) belonging to several compounds of flavonoids in both IBS 3471 and ICPL 87 samples (Table 2, Supplementary Figure S2). Of these, the well-known insect inhibitor, quercetin, was more abundant in IBS 3471 compared to ICPL 87 (Figure 5, Table 2). Other compounds belonging to the flavonol, flavanone, and flavone sub-classes of flavonoids (Table 2) were also observed in both IBS 3471 and ICPL 87. The chromatogram for these compounds in both samples is reported in Supplementary Figure S2. Using the ion compound formula generated and m/z, the compounds identified were probably isorhamnetin belonging to flavonol, naringenin belonging to flavanone, and vitexin or isovitexin and luteolin belonging to flavones. There were multiple retention times (RT) observed for the identified ion formulas and m/z hence, confirmatory experiments to ascertain the flavonoids’ identity is needed.

The quercetin peak area for IBS 3471 was twice that of ICPL 87 (Figure 5, Table 2). All the identified compound peak areas were larger for IBS 3471 than ICPL 87 except for the peak areas for isorhamnetin at 66.6 RT and vitexin or isovitexin, which were larger in ICPL 87 (Table 2).

#### Quercetin Antibiosis Effects on Helicoverpa Armigera Larval Growth and Development

**Figure 6:**
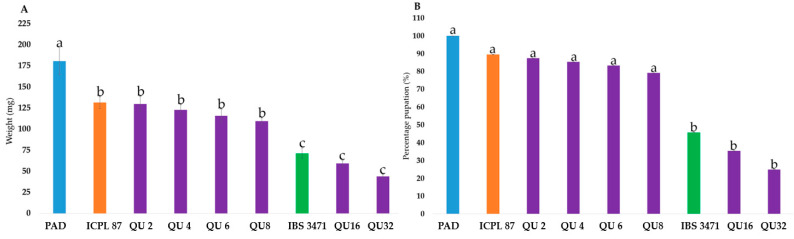
Quercetin antibiosis effect on *H. armigera* larvae. (**A**) Average larval weight in mg observed on Day 3 after setting up the experiment. (**B**) Percentage of pupated *H. armigera* larvae on Day 11 after setting up the experiment. Each bar column represents a different artificial diet larva fed on supplemented with varying concentrations of quercetin (purple bars), lyophilised leaf powder of IBS 3471 (green bar), ICPL 87 (orange bar), and the experimental control, PAD (blue bar). Values labelled with different letters are significantly different at *p* < 0.01 (Tukey’s HSD test). All data are means ± standard errors (*n* = 48). PAD: plain artificial diet and QU: quercetin.

The effects of quercetin (QU), on the development of neonate *H. armigera* larvae were examined using artificial diet supplemented with quercetin at different concentrations (2 mg, 4 mg, 6 mg, 8 mg, 16 mg, and 32 mg per 25 g of dry artificial diet) after three days of larval feeding. The results showed that quercetin inhibited the growth and development of *H. armigera* larvae (Figure 6). Significant differences (F_10,517_ = 45.94, *p* < 0.01) in the weight of *H. armigera* larvae were observed among the artificial diets supplemented with different quercetin concentrations, different lyophilised leaf powders, and plain artificial diet (PAD) (Figure 6A).

The weight of the larvae fed with lyophilised leaves of ICPL 87 was not statistically different (*p* < 0.01) to that of feeding supplemented with QU up to 8 mg/25 g and PAD. Whereas lyophilised leaves of IBS 3471 in PAD diet showed significant inhibition of larval growth similar to the effects of QU16 and QU32 on *H. armigera* larvae (Figure 6A).

By day 11, larvae fed on PAD had 100% pupation, followed by larvae fed on ICPL 87 with 89.6%. There were no significant differences (*p* < 0.01) with pupation percentage among ICPL 87 and QU concentration up to 8 mg/25 g. The highest inhibition and the least pupation observed was on larvae feed on QU32 with 25% pupation on day 11. There were no significant differences (*p* < 0.01) between QU32 and IBS 3471 (Figure 6B).

### 2.5. IBS 3471 Has More Quercetin and Monolignol Biosyntheses Enzymes Than ICPL 87

**Figure 7:**
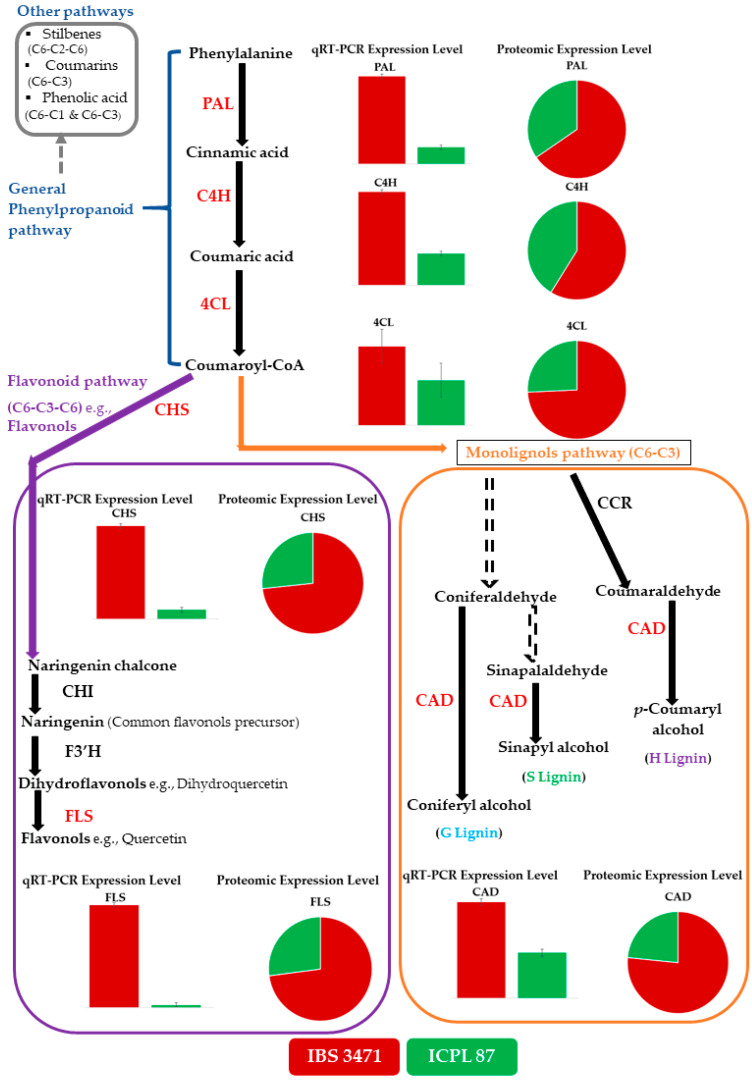
Protein accumulation comparisons with qRT-PCR for general phenylpropanoid, quercetin, and monolignol biosynthesis enzymes between IBS 3471 and ICPL 87. Solid arrows denote single biosynthetic steps while the dashed arrows represent multiple biosynthetic steps. Enzymes investigated are in red font and other enzymes in the pathway are black font. Phenylalanine ammonia-lyase (PAL), cinnamate 4-hydroxylase (C4H), 4-coumarate: CoA ligase (4CL), chalcone synthase (CHS), chalcone isomerase (CHI), flavonoid 3′-hydroxylase (F3′H), flavonol synthase (FLS), cinnamoyl-Coenzyme A reductase (CCR) and cinnamyl alcohol dehydrogenase (CAD). Red shading represent IBS 3471 and green shading is for ICPL 87. Bar graphs represent qRT-PCR expression level and pie chart represent proteomic expression level.

From KEGG metabolism annotation (Figure 3C,D), phenylpropanoid biosynthesis, flavonoid biosynthesis, and flavone and flavonol biosynthesis were among the biosyntheses annotated with more proteins. These are involved in the phenylpropanoid pathway, which results in numerous plant defence compounds such as flavonoids and monolignols. Therefore, quantitative reverse transcriptase-polymerase chain reaction (qRT-PCR) was used to determine the correlation between the differentially abundant proteins (DAPs) and the levels of their corresponding transcribed RNA (highlighted in red font) (Figure 7).

The qRT-PCR analysis showed that the level of transcripts of the selected six enzymes namely, phenylalanine ammonia-lyase (PAL), cinnamate 4-hydroxylase (C4H), 4-coumarate: CoA ligase (4CL), flavonol synthase (FLS), chalcone synthase (CHS), and cinnamyl alcohol dehydrogenase (CAD) were relatively higher in IBS 3471 than ICPL 87 (Figure 7). The pattern for the relative abundance of the selected six enzymes in the phenylpropanoid pathway for IBS 3471 and ICPL 87 from the proteomic analysis was directly correlated with the respective genes’ expression level in the qRT-PCR analysis (Figure 7).

## 3. Discussion

Despite the important role that pigeonpea plays as an economic and food security crop, yields are significantly affected by insect infestation, such as *Helicoverpa armigera* that targets the reproductive structures. As a result, enhancing host plant resistance (HPR) in cultivated crops is crucial in mitigating the insect pests, as *H. armigera* already developed resistance to several common insecticides and some Bt genes [5]. This proteomic analysis provides unique insights into the defence mechanisms of cultivated pigeonpea and one of its CWRs. We performed TMT based quantitative proteomics to analyse the leaf proteomes of two *Cajanus* spp. with contrasting differences in response to *H. armigera* of IBS 3471 (tolerant) and ICPL 87 (susceptible) genotypes. *H. armigera* larvae feed on leaves first before moving to flowers and pods, hence the experiment used the leaves for comparative proteomic analysis. This is among the first proteomic studies done on pigeonpea, and the first using TMT and on *Cajanus scarabaeoides*. TMT labelling allows multiplex relative quantification of proteins in multiple samples [15], which allowed us to test multiple replicated samples from both genotypes at different growth stages simultaneously.

From the comparative proteomic analysis, there were notable differences between the wild and cultivated pigeonpea. The genotypes clustered according to species (*C. scarabaeoides* and *C. cajan*), herbivory response (tolerant or susceptible), and plant growth stages (vegetative, flowering, and podding stage) as expected. These observations are comparable to Kuzina et al. [17], where the winter cress accessions clustered according to their response to flea beetle herbivory (tolerant or susceptible). The wild accession, IBS 3471, tolerant to *H. armigera* had the highest FC of abundant proteins observed at vegetative stage which is the vigorous plant growth stage. Also, at the vegetative stage the plant has the greatest responses to various stresses [18]. A similar pattern of high accumulation of proteins in the wild pigeonpea and clustering of the replicates to their respective species is consistent with previous studies by Njaci et al. [19] and Rathinam et al. [20].

Plants use several mechanisms to respond to tissue injuries. One mechanism is the reallocation of resources to defence-related pathways such as amino acid and sugar metabolism. This mechanism is reflected by the GO classification and KEGG analysis (Figure 3). The results revealed a high abundance of proteins involved in several metabolic pathways, carbohydrate metabolism and biosynthesis of other secondary metabolites and amino acid metabolism, all of which are crucial in plant survival and defence against herbivory. Carbohydrate metabolism provides essential energy for plant survival and defence, while amino acids metabolism is involved in biosynthesis of precursors important for secondary metabolites synthesis. Numerous studies, such as those by Njaci et al. [19], Rathinam et al. [21], Zhou et al. [22] and Rojas et al. [23], have observed that plants mobilise and prioritise metabolomic pathways to produce chemical compounds involved in defence. These include toxic and deterrent compounds, attraction of predator enemies, signalling and triggering an array of physiological responses like ROS generation, systemically acquired response redox homeostasis, and the reconfiguration of primary metabolites compromising growth and reproduction. Our observations are similar to those made by Rathinam et al. [21], using *C. platycarpus,* in which they observed that the wild pigeonpea had more genes involved in secondary metabolite production and cell wall modification when compared with cultivated pigeonpea after *H. armigera* infestation. The increased accumulation of proteins involved in the production of metabolites in response to *H. armigera* may explain the enhanced tolerance in the wild pigeonpea.

Rapid production and accumulation of an oxidative burst is a ubiquitous early plant defence response [24]. Being a rapid response, ROS is also involved in other intricate downstream responses such as the cross-linking of cell wall proteins, induction of defence-related genes, accumulation of phytoalexins, promotion of hypersensitive response, and sometimes triggering programmed cell death [25,26,27]. Besides, ROS affect the insect gut and act as a signalling molecule resulting in upregulation of defence genes [28,29]. After uptake of DAB, the stain reacts with H_2_O_2_ in the plant cells to form a reddish-brown polymer in the presence of peroxidase [30,31]. This defence mechanism contributes to the enhanced tolerance of the wild pigeonpea in response to any form of tissue damage. Meitei et al. [32] observed similar results on DAB staining on pigeonpea leaves where the tolerant pigeonpea leaves of ICPL 332 had darker DAB staining when compared with the leaves of the susceptible ICPL 87 indicating more accumulation of ROS in ICPL 332. Orozco–Cardenas and Ryan, [33] also observed that tolerant tomato had darker DAB staining compared to susceptible tomato.

Secondary metabolites play a defensive role in plants, such as changing the plant’s metabolism, biochemistry and physiology resulting in altered pest behaviour [12,14]. Phenylpropanoid products are involved in crucial plant development and growth responses to biotic and abiotic stress/stimuli [34]. The phenylpropanoid pathway generates several secondary metabolites, including monolignols, flavonoids, phenolic acids, stilbenes, and coumarins [14,35]. The wild pigeonpea, IBS 3471, had higher expression levels of the genes encoding the different enzymes involved in phenylpropanoid pathway than the cultivated pigeonpea, ICPL 87. Transcripts measured by qRT-PCR (Figure 7) correlated with the proteomic results (Figure 7) validating the TMT analysis. Eynck et al. [36], Costa et al. [37], and Lauvergent et al. [38], also reported higher accumulation of monolignol enzymes in tolerant/resistant cultivars. This study is also consistent with the transcriptomic studies of Njaci et al. [19], where there was greater induction of CHS, an important flavonoid enzyme, in the wild, tolerant *C. scarabaeoides* accession in response to *H. armigera* infestation than in the susceptible variety, ICPL 87. Rathinam et al. [39], reported a similar observation in the wild pigeonpea, *C. platycarpus*, with accession having more proteins involved in the phenylpropanoid pathway and flavonoid biosynthesis when compared to a cultivated pigeonpea genotype. Plants appear to reinforce defence against any tissue damage arising either from injuries induced by insects/pathogens or mechanical injuries by reallocating resources and producing secondary metabolites derived from the phenylpropanoid pathway [39,40,41].

So far, there are 27 flavonoids identified in pigeonpea and 14 are found in leaves. They occur in all seven sub-classes and consist of six flavones, two isoflavones, two flavonols, two flavanones, an isoflavanone, and a chalcone [42]. Therefore, leaves are the richest organ for flavonoids in pigeonpea [42]. IBS 3471 the wild pigeonpea had larger peak areas for the identified ion formula and m/z for the possible identified flavonoid compounds compared with ICPL 87 (Figure 5, Table 2). Green et al. [43] observed similar results, where the wild pigeonpea had more flavonoid content than the cultivated susceptible pigeonpea.

The anti-feedant/toxicity properties of many flavonoids, including flavonols such as quercetin, depend on the concentration [44,45]; the higher the amount, the higher the antibiosis effect. Larvae feeding on an artificial diet supplemented with the highest quercetin concentration had the highest larval inhibition on day three (Figure 6A), resulting in delayed growth and development to pupa (Figure 6B). Studies done by Zu et al. [46] quantified cultivated pigeonpea quercetin content to be 0.082 mg/g of dried leaf (i.e., 2 mg/25 g of dry artificial diet, which was the amount used in the bioassay). Several studies have shown that quercetin has an antibiosis effect on several insects including *H. armigera* [47,48] and the silkworm, *Bombyx mori* (L.), [49]. A recent study by Jan et al. [50] reported significant amounts of the flavonols (kaempferol and quercetin), and the anthocyanins (delphinidin and cyanidin), as well as lignin in white-backed planthopper-resistant rice overexpressing a BPH-responsive flavanone 3-hydroxylase (OsF3H) gene compared to the non-transgenic susceptible rice cultivar.

Hence the high accumulation of phenylpropanoid pathway enzymes in IBS 3471 could possibly explain the enhanced tolerance to *H. armigera* while successful transfer of the relevant genes from CWRs to cultivated genotypes may enhance host plant resistance in pigeonpea.

## 4. Materials and Methods

### 4.1. Plant Materials

Seeds of IBS 3471, a *C. scarabaeoides* accession, with high resistance to *H. armigera* larvae [16] and ICPL 87, a high-yielding *C. cajan* variety susceptible to *H. armigera*, [16,51] were obtained from the Australian Grains Genebank (AGG) (Horsham, Victoria, Australia). Mechanically scarified seeds of IBS 3471 and ICPL 87 seeds were germinated and planted in Searles premium potting mix in an environmentally controlled glasshouse (27 ± 1 °C, 13 h light and 11 h dark) at QUT Carseldine facilities, (Brisbane, Queensland, Australia). The leaves were harvested at two stages of the plant growth cycle, the first harvest was during the vegetative growth stage, and the second harvest was during flowering/podding stage (two replicates were at the flowering stage and one was at the early podding stage). The harvested leaves were placed on dry ice and transported to the Centre for Agriculture and the Bioeconomy (CAB) laboratory and stored in −80 °C freezer. To minimise further biochemical composition changes in the leaves, they were freeze-dried for 72 h using Benchtop Pro with Omnitronics™ freeze dryer (VirTis SP SCIENTIFIC, Warminster, PA, USA). Since we used TMT 10 Plex Isobaric label reagent set, we could only accommodate 10 samples in a run. Therefore, two replications for vegetative stage and three replications for flowering/podding stage were included from the two genotypes.

### 4.2. TMT-Based Protein Quantification

#### 4.2.1. Protein Extraction, Reduction, Alkylation and Digestion

Protein quantification using TMT-based proteomics was done as described by Wu et al. [52] and Issacson et al. [53]. Briefly, lyophilised leaf tissue was ground into fine powder in liquid nitrogen, and 50 mg of the crushed leaf powder was resuspended in 10% trichloroacetic acid in acetone (TCA) with 0.07% β-mercaptoethanol before incubation for one hour at −20 °C. The extract was centrifuged at 16,000× *g* for 30 min and the pellet was washed three times, with 1.5 mL of 100% acetone, followed by centrifugation at 16,000× *g* for 30 min. The collected pellet was dried in a vacuum centrifuge before being resuspended in 2% sodium dodecyl sulphate (SDS) in 8 M urea, 100 mM Tris-HCL (pH 8.8), followed by reduction and alkylation with 10 mM dithiothreitol (DTT) and 20 mM iodoacetamide (IAM). The collected protein pellet was reconstituted with 8 M urea, 100 mM Tris-HCL (pH 8.8) in water. Protein concentration was measured using bicinchoninic acid assay (BCA assay) (Thermo Fisher Scientific, Waltham, MA, USA) as per the manufacturer’s protocol. Sample proteolysis with Lys-C (100:1 protein to enzyme ratio) was done overnight at 28 °C, followed by digestion with trypsin (100:1 protein to enzyme ratio) at 37 °C for six hours. The pH of the samples was adjusted to three using a final concentration of approximately 1% trifluoroacetic acid (TFA), before desalting using a solid-phase extraction disc styrene divinyl benzene containing stage tips (Empore SDB-RPS 47 mm extraction disc, Merck KGaA, Darmstadt, Germany). Stage tips were self-packed into pipette tips, while peptides were bound to the stage-tip washed with 0.2% TFA and before elution with 80% acetonitrile (ACN): 5% ammonium hydroxide (NH_4_OH). The eluted peptides were dried by centrifugation under vacuum. The dried pellet was reconstituted in 200 mM HEPES pH 8.8, and the concentration of the peptide was determined using Pierce quantitative colourimetric peptide assay (Thermo Fisher Scientific, Waltham, MA, USA).

#### 4.2.2. TMT Sample Labelling

TMT reagent labelling of peptides (Thermo Fisher Scientific, Waltham, MA, USA) was done according to the manufacturer’s protocol as follows. For each TMT label vial, anhydrous acetonitrile was added before vortexing for five minutes, followed by brief centrifugation. Aliquots of each different peptide samples were labelled with one of the individual ten TMT labels at room temperature for one hour with occasional vortexing (Table 3). The excess TMT label in the sample was quenched with the addition of 5% hydroxylamine, vortexed, and incubated at room temperature for 15 min.

Labelled peptides were pooled after a label check experiment was done by mixing 1.5 μL of each individually labelled TMT sample. The label check ensured equal volumes of total peptides were pooled from all the samples. After a normalisation factor was obtained from the label check experiment, the TMT-labelled peptide samples were pooled at a 1:1 ratio across all samples and vacuum dried. Desalting was done using C18 solid-phase extraction (SPE, Sep-Pak, Waters, Helsinki, Finland) and the cleaned samples were vacuum centrifuged until dry. High pH (HpH) reverse-phase High-Performance Liquid Chromatography (HPLC) was used to fractionate the peptide mixture into 96 fractions, which were consolidated into 17 fractions before LC-MS/MS analysis.

#### 4.2.3. Data Acquisition Using Nanoflow Liquid Chromatography-Electrospray Ionisation-Tandem Mass Spectrometry (NanoLC ESI MS/MS)

The HpH HPLC fractions of each TMT set were reconstituted with sample loading buffer (2% ACN, 97.9% water, 0.1% Formic acid [FA]). The samples were subjected to LC-MS/MS (Easy nLC 1000, Thermo Fisher Scientific, Waltham, MA, USA) analysis, and 1D Data-dependent acquisition (DDA) of peptides was done on Q-Exactive Quadrupole-Orbitrap (QE-Classic, Thermo Fisher Scientific, Waltham, MA, USA). The TMT labelled peptides samples were injected onto an in-house packed trap column (Halo C18, 160 Å, 2.7 μm, 100 μm × 3.5 cm) and desalted with loading buffer (99.9% water, 0.1% FA). An in-house packed analytical column (Halo C18, 160 Å, 2.7 μm, 75 μm × 15 cm) with a linear gradient of mobile phase A (99.9% water, 0.1% FA) and mobile phase B (99.9% ACN, 0.1% FA): mobile phase B (30%) over 110 min with a flow rate of 300 nL/min across the gradient was used to elute peptides from the trap before they were separated over the analytical column. The eluent from the column was directed into a 2.6 kV electrospray voltage from the mass spectrometer ionisation source via a liquid junction upstream of the column. An automatic gain control (AGC) target value of 1 × 10^6^ was used to scan at 70 k resolution for peptide precursors from 350 to 1850 m/z. Higher-Energy Collisional Dissociation (HCD) using a normalised collision energy of 35 with an isolation width of 0.7 m/z was used to fragment the ten most intense ions from the previous survey scan. MS/MS analysis was done for precursors with charge state +2 to +4. For the MS2, the minimum signal required was 2.5 × 10^4^, an AGC target value of 2 × 10^5^ and a maximum injection time of 250 ms. MS/MS scan resolution was 70 k, and the dynamic exclusion was 90 s.

#### 4.2.4. Protein Identification and Quantification

Proteome Discoverer software version 2.1 (Thermo Fisher Scientific, Waltham, MA, USA) was used to search mass spectrometric data files generated from Xcalibur™ software (Thermo Fisher Scientific, Waltham, MA, USA). SequestHT and Mascot (Matrix Science, London, UK) search engines were used to process the data against *Cajanus cajan* and *Cajanus scarabaeoides* sequences downloaded from the Uniprot database (47,669 sequences, accessed on 19 July 2020 [54]).

Trypsin enzyme with two maximum missed cleavages, with a 20 ppm precursor mass tolerance and 0.02 Da fragment mass tolerance, was used for peptide identification and quantification. The parameters used for dynamic modifications were oxidation of methionine, deamination of asparagine, glutamine and pyroglutamate, acetylation of protein N-terminus, Met-loss+Acetyl (Sequest) and TMT6plex tag on lysine residues, and the peptide N-terminus. Static modification parameter was carbamido-methylation of cysteine. False discovery rate (FDR) was set at <1%, while the display filters were Protein, Peptide, and PSM Master Proteins only.

#### 4.2.5. Gene Ontology (GO) Classification

The Gene Ontology (GO) classification was done via the PANTHER (protein annotation through evolutionary relationship) (University of Southern California, Los Angeles, CA, USA) classification system [55] grouping into the molecular function (MF), cellular component (CC), and biological process (BP). Further annotation was done using Kyoto encyclopedia of genes and genomes (KEGG) BlastKOALA (KEGG Orthology And Links Annotation), (Kyoto University, Kyoto, Japan) [56].

### 4.3. DAB Staining Analysis

The oxidative burst in the leaves was examined by 3,3′-Diaminobenzidine (DAB) staining (Sigma-Aldrich, D8001, CAS # 91-95-2, Merck KGaA, Darmstadt, Germany) as described by Daudi and O’Brien [57]. Briefly, the punched leaf discs from both IBS 3471 and ICPL 87 were placed in 2 mL of DAB staining solution in a Petri dish, while for the controls, the leaf discs were placed in 2 mL of 200 mM disodium phosphate (Na_2_HPO_4_). The Petri dishes were covered with aluminium foil and placed on a shaker at 90 rpm for five hours. The leaf discs were transferred into falcon tubes with bleaching solution (3:1:1 Ethanol: Acetic acid: Glycerol) and placed on a heating block at 90 °C for 15 min. The bleaching solution was changed with flesh bleaching solution and allowed to stand for 30 min at room temperature. The leaf discs were patted dry and imaged.

### 4.4. Identification of Flavonoids Compounds Using Liquid Chromatography-Mass Spectrometry (LCMS)

The freeze-dried leaves for IBS 3471 and ICPL 87 (same batch used for proteomic analysis at the vegetative stage) were milled, using a Tube Mill Control (IKA Mills No. 0004180000, Guangzhou, China) for 5 s, at 25,000 rpm. Flavonoid extraction was done using two grams of freeze-dried leaves powder with 40 mL HPLC grade Methanol (Sigma-Aldrich, 34860, CAS # 67-56-1, Merck KGaA, Darmstadt, Germany) in an ultrasonic water bath (Elmasonic P Series, Elma, Singen, Germany) at a frequency of 37 KHz at 30 °C for 10 min [58].

The extract was squeezed through cheesecloth before filtration using 0.22µm Hydraflon™ membrane filter (MicroScience cat # MS SF13HY022, New South Wales, Australia). The filtered extracts were vacuum dried at room temperature and reconstituted with 4 mL of HPLC grade Methanol. Three µL of the extracts were injected onto an Orbitrap Elite mass spectrometer equipped with a heated electrospray ionisation (ESI) source operating in the negative ion mode.

Mass spectra were acquired by scanning from m/z 80–750 at a mass resolution of 240,000 (defined at m/z 400). Nitrogen was used as the sheath and auxiliary gas at 30 and 10 arbitrary units, respectively. The spray voltage was 3 kV, and the source heater temperature was 260 °C, and the capillary temperature was 320 °C. Solvent A was 1% acetic acid in water and solvent B was methanol and the gradient used was 0–1.4 min: 2% B, 1.4–8.4 min: 2–5% B, 8.4–47.6 min: 5–20% B, 47.6–63.0 min: 20–50% B, 63.0–67.2 min: 50–55% B, 67.2–71.4 min: 55–2% B and 71.4–85.4 min: Re-equilibrated with 2% B. The flow rate was 0.5 mL/min, and the column temperature was 27 °C. The column used for the analysis was C18 XP Column, 130 Å, 2.5 µm, 3 mm × 100 mm, (Waters XBridge BEH, Part # 186006035, Helsinki, Finland). Mass spectrometric data files from the Orbitrap Elite mass spectrometer were generated using Xcalibur™ software (Thermo Fisher Scientific, Waltham, MA, USA).

### 4.5. Biological Assays: Screening For H. Armigera Resistance (Antibiosis Mechanism) Using Artificial Diet Supplemented with Different Quercetin Concentrations Bioassay

*H. armigera* egg colonies obtained from the Commonwealth Scientific and Industrial Research Organisation (CSIRO), Narrabri, (New South Wales, Australia) were hatched at 25 °C.

The artificial diet was prepared by adding Stonefly Heliothis diet (Ward’s ScienceX) powder (25 g), 100 mL of water-vinegar (19:1), and quercetin (Sigma-Aldrich, Q4951, CAS # 117-39-5, Merck KGaA, Darmstadt, Germany) at concentrations of 2 mg, 4 mg, 6 mg, 8 mg, 16 mg, and 32 mg per 25 g of dry artificial diet. The lyophilised leaf powder for IBS 3471 and ICPL 87 was prepared as described in Ngugi–Dawit et al. [16]. In brief, 3.3 g of lyophilised leaf powder was mixed with 21.7 g of Stonefly Heliothis diet (Ward’s ScienceX, Rochester, NY, USA) powder and 100 mL of water-vinegar (19:1). A plain artificial diet (PAD) without quercetin and lyophilised leaf powder was used as a control.

The neonate *H. armigera* larvae were placed into each well of a 32-cell rearing tray (RT32W, Frontier Scientific Services Agriculture, Newark, DE, USA) using a moist paintbrush. The sealed rearing trays were placed in a Sanyo Versatile environmental test chamber (Model MLR-350H, Osaka, Japan) at 26 ± 1 °C, 16 h light and 8 h dark. Each treatment had 48 replications and the larval weights were recorded after three days.

### 4.6. Confirmatory and Validation Analysis of Protein Expression Using qRT-PCR Analysis

Quantitative reverse transcriptase-polymerase chain reaction (qRT-PCR) was carried out on selected genes to determine the correlation between the expression levels of genes and protein abundance obtained from TMT proteomic analysis.

Total RNA was extracted using RNeasy^®^Plant mini kit (QIAGEN cat # 74903, Victoria, Australia) following the manufacturer’s protocol. DNA contamination from the samples was removed with on-column DNase treatment during extraction using RNase-Free DNase set (QIAGEN cat # 79254, Victoria, Australia) and post-extraction using RQ1 RNase-Free DNase (Promega cat # M6101). Complementary DNA (cDNA) synthesis was done using M-MLV Reverse Transcriptase (Promega part # M170A, Madison, WI, USA) according to the manufacturer’s protocol.

qRT-PCR was done using GoTaq^®^ qPCR MasterMix (Promega Corporation, cat # A6001, Madison, WI, USA) on BIO-RAD C1000Touch™ Thermal Cycler (Model CFX384™ Real-Time System, Singapore) machine. The thermal cycle profile was: 95 °C for 3 min, 45 cycles at 95 °C for 10 s, and 60 °C for 30 s followed by a melting profile at 95 °C for 10 s, 65 °C for 5 s, and 95 °C for 5 s. Each sample had three biological and three technical replicates. The relative fold changes were calculated using the 2^−ΔΔCt^ method as described by Livak and Schmittgen [59]. Actin 1 and Initiation factor 4α (IF4α) were used as the reference genes. All the primers used in this study are listed in Supplementary Table S2.

### 4.7. Data and Statistical Analysis

The generated proteomic output from the Proteome Discover software was analysed using TMTPrepPro package (Macquarie University, Sydney, New South Wales, Australia) [60] for proteomic statistical analysis and unsupervised clustering analyses. Proteomic statistical analysis parameters were *p* < 0.05 with a fold change of ±1.5, and all the proteins with missing values were removed before the analysis. The adjusted *p*-value was done using the Benjamini–Hochberg (BH) adjustment analysis. TMTPrepPro package was also used to generate boxplots, density plots, volcano plots, heat-map, and 3 D PCA loading and scores. Boxplots, density plots, and volcano plots were generated using all identified high confident protein, whereas for the heat map and PCA, they were generated using the differentially abundant proteins (DAPs).

The feeding bioassay data were analysed using Minitab^®^ Statistical Software Version 18.1, (State College, Pennsylvania, USA). Analysis of variance (one-way ANOVA) and post hoc mean comparisons with Tukey’s HSD test at *p* < 0.01 was conducted for all parameters, comparing multiple means. Parameters comparing two means were analysed, using the independent *t*-test (the *p*-value of a Student’s *t*-test < 0.01). Results are presented as means ± SE.

## 5. Conclusions

In conclusion, the present study reveals that the enhanced tolerance to *H. armigera* in IBS 3471 is related to the high fold change (FC) of the identified DAPs being involved and is crucial in plant defence and response mechanisms such as ROS scavenging (antioxidants), the phenylpropanoid pathway, signalling, and transduction pathways. Consequently, this confers superior defence mechanisms to IBS 3471 when compared to *C. cajan* ICPL 87. Therefore, this study has given an insight into the enhanced *C. scarabaeoides* insect resistance and defence mechanisms, laying the foundation for further studies. IBS 3471 is an ideal candidate for improving host plant resistance mechanism and expanding the genetic base in cultivated pigeonpea.

## Figures and Tables

**Table 1:** A representative subset of DAPs involved in plant defence selected for further studies from top DAPs. (The complete list of proteins is provided in Supplementary Table S1).

UniProt ID	Protein Description	Proteomic LOG_2_FC	ANOVA (*p* < 0.05)	Role
Antioxidant, ROS Scavenger & Detoxification Enzymes				
A0A151R1K6	Putative glutathione S-transferase	5.674	0.000	Antioxidant/ ROS scavenger/detoxifying enzyme
A0A151RS05	Cytochrome P450	3.688	0.000	Biosynthesis of primary & secondary metabolites
A0A151QVW9	Peroxidase	2.591	0.010	Response to stress/Antioxidant
A0A151TXJ4	Superoxide dismutase [CuZn]	2.567	0.002	Scavenging of O_2_ radicals/superoxide anions
Secondary Metabolite				
A0A151QU72	Anthocyanidin 5,3-O-glucosyltransferase	2.898	0.001	Anthocyanin biosynthesis pathway
A0A151RR63	Isoflavone-7-O-methyltransferase	2.496	0.001	Phytoalexin biosynthesis
A0A151RRX4	Dirigent protein	2.007	0.010	Monolignol pathway
Signalling				
A0A151U2F2	Calcium-dependent protein kinase	2.714	0.004	Signal transduction pathways
A0A151U2V3	Hydroxyethylthiazole kinase	2.538	0.002	Cofactor biosynthesis pathway
A0A151TPW7	Annexin	1.810	0.003	Calcium-dependent membrane-binding proteins
A0A151SDR5	Jasmonate O-methyltransferase	3.156	0.000	Signalling & defence-related processes
Anti-Nutritional Factors				
A0A151SAI2	Lysosomal Pro-X carboxypeptidase	3.160	0.009	Herbivore defensive protein
A0A151RBF5	Subtilisin-like protease	2.256	0.000	Serine proteases (development & signalling)
A0A151QZF6	Cysteine proteinase inhibitor	2.343	0.001	Herbivore defensive protein
Carbohydrate Metabolism				
A0A151QPT9	Acidic endochitinase	3.824	0.009	Response to biotic & abiotic stresses
A0A151TII8	Lipase	2.999	0.002	Synthesis of signalling defence lipids & precursors
Phytohormone				
A0A151RG77	Cytokinin-Oglucosyltransferase	3.730	0.000	Cytokinin (biotic & abiotic stress responses)
A0A151RKG5	1-aminocyclopropane-1-carboxylate oxidase	1.812	0.001	Ethylene (development & stress responses)

**Table 2:** Identified flavonoids in IBS 3471 and ICPL 87 using LCMS.

Flavonoid Class	Possible Compounds	Ion Formula (Negative Ion Mode)	Mass-To-Charge (M/Z)	Retention Time (Minutes)	Peak Area		Fold Change (IBS 3471/ICPL 87)
					IBS 3471	ICPL 87	
Flavonols	Quercetin	C_15_H_9_O_7_^−^	301.0354	62.5	1,774,965	952,200	2
	Isorhamnetin	C_16_H_11_O_7_^−^	315.051	64.6	450,240	114,517	4
				66.6	146,742	1,356,558	0
Flavanones	Naringenin	C_15_H_11_O_5_^−^	271.0612	55.9	273,446	35,715	8
				63.1	180,120	33,276	5
				64.6	27,846	6202	4
Flavones	Vitexin or Isovitexin	C_21_H_19_O_10_^−^	431.0984	55.8	61,411,155	61,736,130	1
	Luteolin	C_15_H_9_O_6_^−^	285.0405	63.8	3,130,076	523,375	6
				65.8	1,687,625	145,539	12

**Table 3:** Details of the TMT reagent labelling of peptides used for each IBS 3471 and ICPL 87 samples analysed under different growth stages.

Sample Name	Replication	Growth Stage	TMT Reagent Labelling of Peptide
IBS 3471 ^1^ (*Cajanus scarabaeoides*)	Rep 1	Vegetative stage	126
	Rep 2		127N
	Rep 1	Flowering/podding stage	127C
	Rep 2		128N
	Rep 3		128C
ICPL 87 ^2^ (*Cajanus cajan*)	Rep 1	Vegetative stage	129N
	Rep 2		129C
	Rep 1	Flowering/podding stage	130N
	Rep 2		130C
	Rep 3		131

^1^ tolerant to *H. armigera*, ^2^ susceptible to *H. armigera.*

## Data Availability

The data supporting the findings of this study are available online at http://doi.org/10.5281/zenodo.4724395.

## Supplementary Materials

The following are available online at https://www.mdpi.com/article/10.3390/ijms22115941/s1. Table S1: List of identified proteins in the study, Figure S1: Post-normalised data showing variability in the TMT-MS data. Figure S2: LCMS zoomed chromatogram of identified flavonoids compounds, Table S2: List of primers used for qRT-PCR in the study.
