# Supplementary material for: Comparative TMT Proteomic Analysis Unveils Unique Insights into Helicoverpa armigera (Hübner) Resistance in Cajanus scarabaeoides (L.) Thouars
Source: Int J Mol Sci. 2021 May 31;22(11):5941. doi: 10.3390/ijms22115941 (PMC8198728; doi:10.3390/ijms22115941)
Supplement: Supplementary file 1 [file ijms-22-05941-s001.zip › Supplementary materials_Comparative TMT proteomics manuscript/Supplementarly Figure S1,S2& tableS2_Comparative TMT proteomics_Ngugi-Dawit et al.pdf]

International Journal of
Molecular Sciences

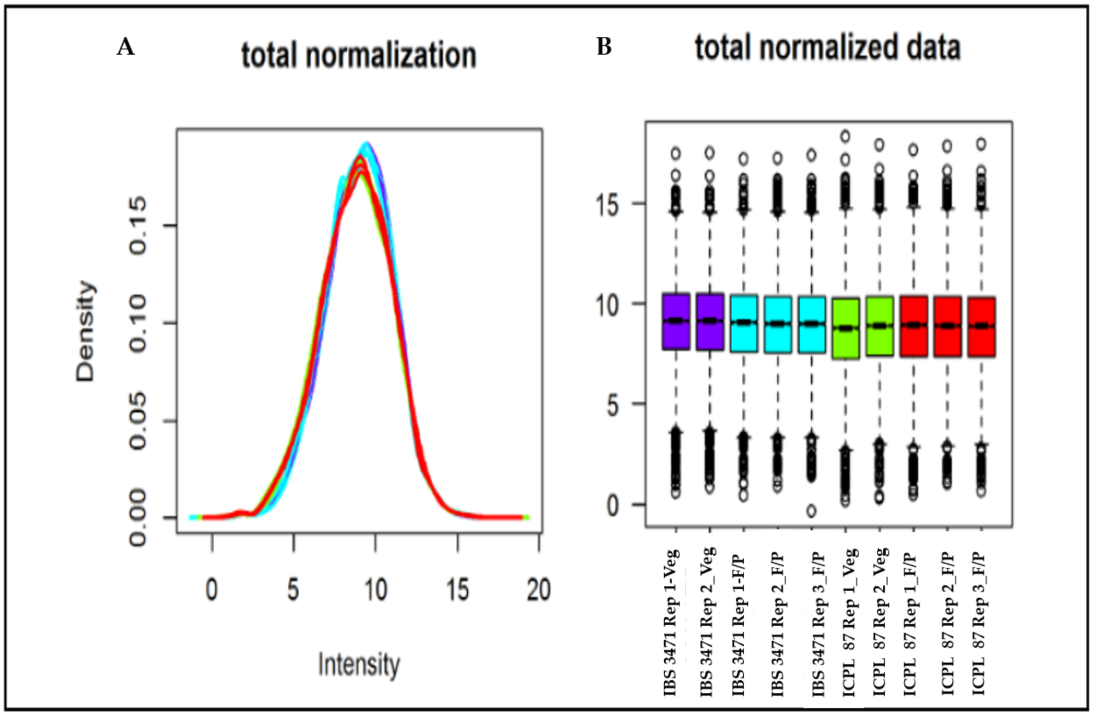

Supplementary Figure S1. Post-normalised data showing variability in the TMT-MS data. (**A**) Density plot and (**B**) Box plots of all identified proteins. ICPL 87 at flowering/podding stage [replication 3] was used as reference ratio across all samples.

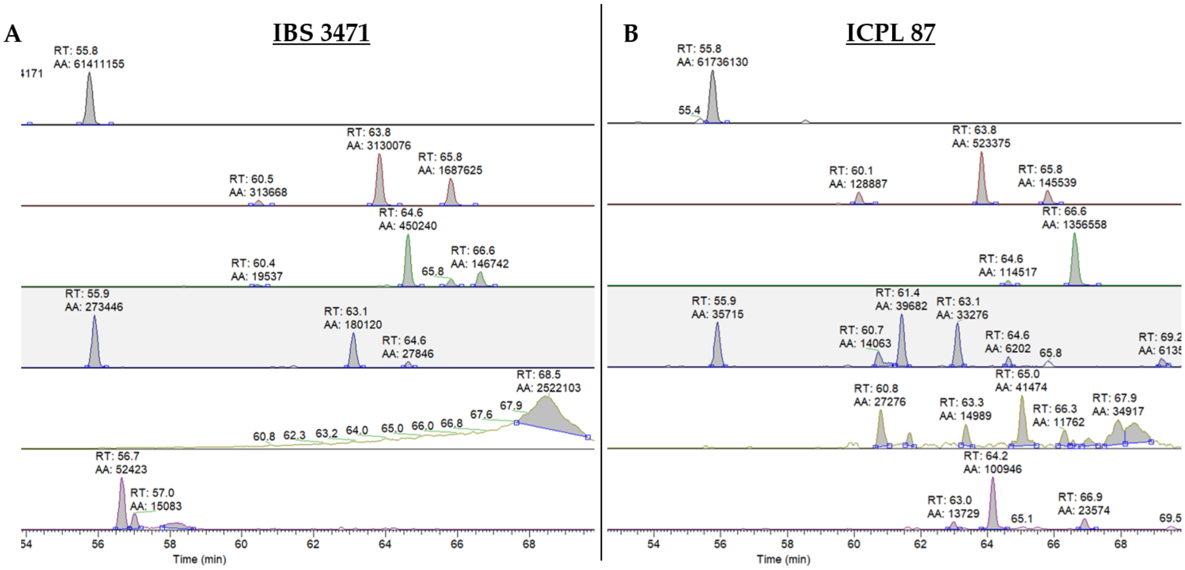

Supplementary Figure S2. LCMS zoomed chromatogram of identified flavonoids compounds. (**A**) IBS 3471 chromatograms and (**B**) ICPL 87 chromatogram.

Supplementary Table S2. List of primers used for qRT-PCR in the study

Pathway	Gene ID	Primer type	Primer sequence 5' - 3'	Amplicon size (bp)	Source
General Phenylpropanoid pathway	Phenylalanine ammonia-lyase (PAL)	F-	CTTGCCCTTGTGAATGGCAC	92	This study
		R-	AKAACTTCAGACAAGACAGC		
	Cinnamate 4-hydroxylase (C4H)	F-	CTCCTCCTGGCCTCAACAAG	100	
		R-	AACGACCTTGGCTTTGCAAC		
	4- Coumaroyl-CoA ligase (4CL)	F-	TGGTGGCTCTGACGAAAGAC	97	
		R-	AAGACTTCCTTTCCCAGCGG		
Flavonol biosynthesis	Chalcone synthase (CHS)	F-	GAYGAGATGAGAAGGTGGTC	95	
		R-	GTYAAKCCAGGGCCAAACCC		
	Flavonol synthase (FLS)	F-	TCAACTACGACATGTGGCCC	123	
		R-	CCAACCCTAACCCTTCGGAC		
Monolignols biosynthesis	Cinnamyl alcohol dehydrogenase (CAD)	F-	GCTCCTCTACTATGCGCAGG	117	
		R-	AGCTATGTGTCCAAGCCCAC		
House Keeping Genes (Reference genes)	Initiation factor 4α	F-	GCCGAGATCACACAGTCTCA	95	Sinha et al. 2015
		R-	ACCACGAGCCAAAAGATCAG		
	Actin 11	F-	GGCATACATTGCCCTTGACT	97	
		R-	GAACCTCGGGACATCTGAAA		
